# The NEDD8-activating enzyme inhibitor MLN4924 sensitizes a TNFR1^+^ subgroup of multiple myeloma cells for TNF-induced cell death

**DOI:** 10.1038/s41419-019-1860-2

**Published:** 2019-08-13

**Authors:** Mohamed El-Mesery, Tina Rosenthal, Hilka Rauert-Wunderlich, Martin Schreder, Thorsten Stühmer, Ellen Leich, Andreas Schlosser, Martin Ehrenschwender, Harald Wajant, Daniela Siegmund

**Affiliations:** 1 Department of Biochemistry, Faculty of Pharmacy, Mansoura University, Mansoura, Egypt; 2 Division of Molecular Internal Medicine, Department of Internal Medicine II, University Hospital Würzburg, Auvera Haus Grombühlstraße 12, 97080 Würzburg, Germany; 3 Institute of Pathology, University of Würzburg, Josef-Schneider-Straße 2, 97080 Würzburg, Germany; 4 Comprehensive Cancer Center Mainfranken, University Hospital Würzburg, 97080 Würzburg, Germany; 5 Lehrstuhl für Translationale Onkologie, Comprehensive Cancer Center Mainfranken, University Hospital Würzburg, Versbacher Straße 5, 97078 Würzburg, Germany; 6 Rudolf Virchow Center for Experimental Biomedicine, University of Würzburg, Josef-Schneider-Straße 2, 97080 Würzburg, Germany; 7 Institute of Clinical Microbiology and Hygiene, University Hospital Regensburg, Franz-Josef-Strauss-Allee 11, 93053 Regensburg, Germany

**Keywords:** Tumour-necrosis factors, Cancer therapy

## Abstract

The NEDD8-activating enzyme (NAE) inhibitor MLN4924 inhibits cullin-RING ubiquitin ligase complexes including the SKP1-cullin-F-box E3 ligase βTrCP. MLN4924 therefore inhibits also the βTrCP-dependent activation of the classical and the alternative NFĸB pathway. In this work, we found that a subgroup of multiple myeloma cell lines (e.g., RPMI-8226, MM.1S, KMS-12BM) and about half of the primary myeloma samples tested are sensitized to TNF-induced cell death by MLN4924. This correlated with MLN4924-mediated inhibition of TNF-induced activation of the classical NFκB pathway and reduced the efficacy of TNF-induced TNFR1 signaling complex formation. Interestingly, binding studies revealed a straightforward correlation between cell surface TNFR1 expression in multiple myeloma cell lines and their sensitivity for MLN4924/TNF-induced cell death. The cell surface expression levels of TNFR1 in the investigated MM cell lines largely correlated with TNFR1 mRNA expression. This suggests that the variable levels of cell surface expression of TNFR1 in myeloma cell lines are decisive for TNF/MLN4924 sensitivity. Indeed, introduction of TNFR1 into TNFR1-negative TNF/MLN4924-resistant KMS-11BM cells, was sufficient to sensitize this cell line for TNF/MLN4924-induced cell death. Thus, MLN4924 might be especially effective in myeloma patients with TNFR1^+^ myeloma cells and a TNF^high^ tumor microenvironment.

## Introduction

Multiple myeloma (MM) is a malignancy of differentiated blood B-cells. This kind of malignancy localizes to the bone marrow leading to osteolysis, bone pain and impaired hematopoiesis^1^. Several research trials have been directed to treat MM and the current treatment strategies include chemotherapy and stem cell transplantation^2^. A class of chemotherapeutic drugs which are particular successful in the treatment of MM are proteosome targeting drugs, such as bortezomib and carfilzomib^3^.

The homodimeric and heterodimeric transcription factors of the NFκB family fulfill a variety of functions in different biological processes including immune response, development, cell growth and cell survival. Activation of NFκB transcription factors depends on proteolytic degradation/processing of IκB proteins or proteins containing an IκB-related domain that hinder nuclear translocation of NFκB transcription factors by intermolecular interaction or intramolecular binding, respectively^4,5^. Activation of NFκB transcription factors occurs via two distinct pathways: the classical and the alternative NFκB pathway^4,5^. The classical NFκB pathway depends on activation of the IκB kinase (IKK) complex, e.g., by receptors of the tumor necrosis factor superfamily (TNFRSF) or pattern recognition receptors, IKK-mediated phosphorylation of IκBs, K48 ubiquitination of phospho-IκBs by the E3 ligase β-transducin repeat containing protein (βTrCP) and eventually proteasomal degradation of phosphorylated IκBs. The alternative pathway is primarily triggered by a subset of receptors of the TNFRSF and includes phosphorylation of the IκB-related domain containing NFκB precursor protein p100 by IKK1, phosphorylation of phospho-p100, K48 ubiquitination of phospho-p100, again by the E3 ligase βTrCP, and proteasomal p100 processing to the NFκB subunit p52^6^.

A variety of stimuli activate the classical NFκB pathway, including physical stressors such as UV light but also receptors of the TNF receptor superfamily or pattern recognition receptors^4,5^. TNFR1 is one of the strongest activators of the classical NFκB pathway and can be activated by both soluble and membrane TNF^7^. On the other hand, the second known TNF receptor, TNFR2, induces mainly the alternative NFκB pathway with only moderate activation of the classical NFκB pathway^8^. MM is characterized by elevated TNF expression and a deregulated NFκB system^9,10^.

The small molecule MLN4924 (Pevonedistat) inhibits the NEDD8-activating enzyme (NAE) which neddylates the cullin subunit of cullin RING E3 ligases (CRLs). Therefore, this drug acts indirectly as an inhibitor of CRLs, too. Since the βTrCP E3 ligase is a CRL^11,12^, MLN4924 acts also as an inhibitor of the two NFĸB pathways by prevention of proteasomal degradation of IκBα and p100 processing^13–16^. CRLs and NAE are often particularly active in cancer cells and promote growth, survival and invasion of tumor cells. In line with this, in vitro and in vivo studies revealed anti-tumoral activity of MLN4924 in a variety of cancer models and several clinical phase I studies indicate that MLN4924 is well tolerable^17,18^. At the molecular level, the anti-tumoral effects of MLN4924 have been traced back to the inhibition of the proteasomal degradation of various negative regulators of the DNA damage response, the cell cycle and senescence^17^. Recently, it was proved by us and others that MLN4924 enhances TNF-induced cell death^19,20^. As far as proteasome targeting drugs are successfully used to treat MM, an alternative strategy to target the ubiquitin-proteasome pathway in MM might lay in the inhibition of NAE by MLN4924. Therefore, we investigated here the effect of TNF and MLN4924 on the viability of MM cell lines and primary myeloma cells. MM cells were found to be generally TNF resistant but MLN4924 sensitized a subset of myeloma cells for TNFR1-induced cell death. It turned out that cell surface expression of TNFR1 is decisive for TNF/MLN4924 sensitivity.

## Results

### MLN4924 is less cytotoxic than carfilzomib and bortezomib on multiple myeloma cell lines

**Fig. 1:**
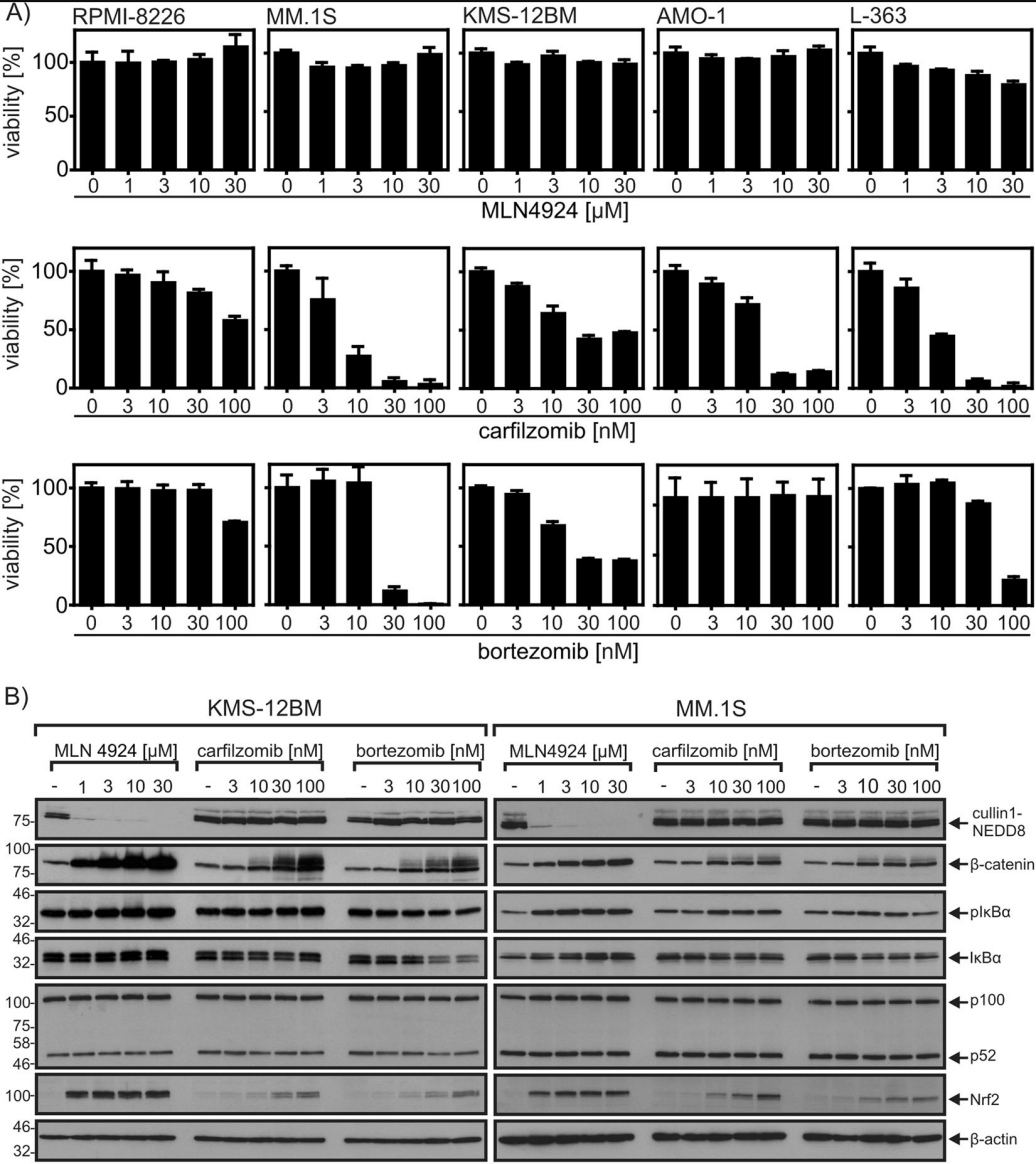
Accumulation of CRL-regulated proteins in MLN4924-treated, carfilzomib-treated, and bortezomib-treated multiple myeloma cell lines. **a** Cells were challenged in technical triplicates for 24 h with the indicated concentrations of MLN4924, carfilzomib and bortezomib. Cell viability was determined using the MTT assay. **b** Cells were treated as indicated with MLN4924, carfilzomib, and bortezomib and total cell lysates were then analyzed for the presence of the indicated proteins by Western blotting. Data shown are representative of at least two independent experiments

**Fig. 2:**
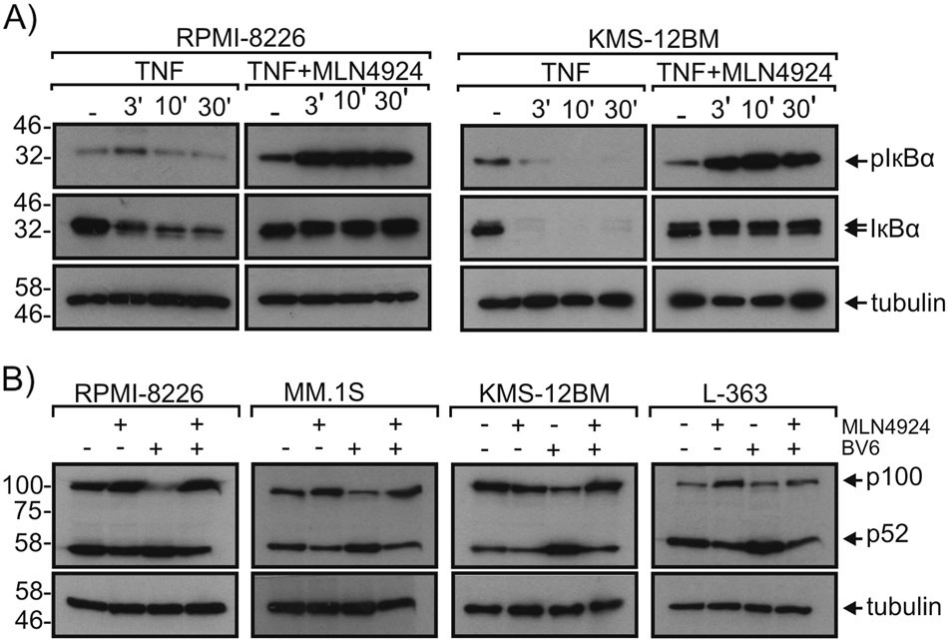
MLN4924 inhibits TNF-induced and BV6-induced NFκB signaling. **a** RPMI-8226, MM.1S, and KMS-12BM cells were stimulated with 100 ng/ml TNF for the indicated times in the presence and absence of 20 µM MLN4924. Total cell lysates were analyzed for phosphorylation and degradation of IκBα. **b** The indicated cell lines were treated overnight with 10 µM of the SMAC mimetic BV6 in the presence and absence of 20 µM MLN4924 and total cell lysates were analyzed for p100 processing. Data shown are representative of at least two independent experiments

Initially, we compared the cytotoxic effects of MLN4924, carfilzomib and bortezomib on the five human MM cell lines RPMI-8226, MM.1 S, KMS-12BM, AMO-1, and L-363. While bortezomib and carfilzomib induced in nearly all cell lines significant to high cell death within 24 h, MLN4924 had no or only a minor cytotoxic effect (Fig. 1a). The lack of acute cytotoxicity of MLN4924 was not related to missing or suboptimal drug activity. The range of MLN4924 concentrations evaluated included doses ensuring maximal inhibition of NAE and efficient accumulation of CRL substrates such as β-catenin (Fig. 1b). Indeed, the accumulation of these substrates in MLN4924-treated cells was even higher and more efficient than in bortezomib-treated or carfilzomib-treated cells. Mass spectrometric analysis furthermore revealed accumulation of various well-established CRL substrates in MLN4924-treated myeloma cells (Table 1). The MLN4924 doses used were also fully sufficient to abrogate TNF-induced activation of the classical NFκB pathway. This was evident from western blot analyses showing accumulation of phospho-IκBα and abrogated IκBα degradation (Fig. 2a). Likewise, MLN4924 strongly inhibited SMAC mimetic-induced processing of the CRL substrate p100, a crucial step in the activation of the alternative NFκB pathway (Fig. 2b).

**Table 1:** MLN4924-induced accumulation of proteins observed by SILAC mass spectrometry

Gene name of accumulated protein	Log 2 protein expression MLN4924 treated/untreated				
	INA6	KMS11	L363	MM1.S	OPM2
CCND3	n.d.	3,62	2,25	0,60	n.d.
CCND2	n.d.	2,57	3,80	2,47	2,79
CDKN2A	1,87	3,58	3,84	n.d.	3,39
CDT1	n.d.	2,26	3,03	3,22	2,82
CTNNB1	2,96	NA	2,25	4,07	1,00
ETNK1	0,15	1,69	2,46	2,18	0,88
FBXO28	n.d.	n.d.	1,87	2,06	1,95
FEM1B	n.d.	n.d.	3,00	1,73	n.d.
FYTTD1	1,25	1,10	0,95	2,17	1,17
HMOX1	2,05	3,70	0,65	−0,07	2,16
KLHL6	0,70	n.d.	2,00	2,38	n.d.
LRRC58	1,48	2,86	3,06	3,38	3,35
MCM10	n.d.	2,46	n.d.	2,91	n.d.
MORF4L1	1,45	0,87	1,79	2,15	1,50
MORF4L2	1,69	1,18	2,11	2,34	1,91
MRFAP1	2,10	2,87	2,45	0,61	2,74
NUSAP1	0,09	1,02	1,36	2,38	0,82
SHKBP1	n.d.	2,13	2,00	n.d.	n.d.
SRXN1	1,21	2,68	2,16	2,87	2,60
TRCP4AP	n.d.	n.d.	3,56	3,24	n.d.
TSPYL1	1,20	n.d.	n.d.	2,18	0,69

Factors are listed which were upregulated in at least two MM cell lines with log 2 > 1 and at least once with log 2 > 2. *n.d.* not detected

### MLN4924 sensitizes a subset of myeloma cell lines for TNFR1-induced cell death

**Fig. 3:**
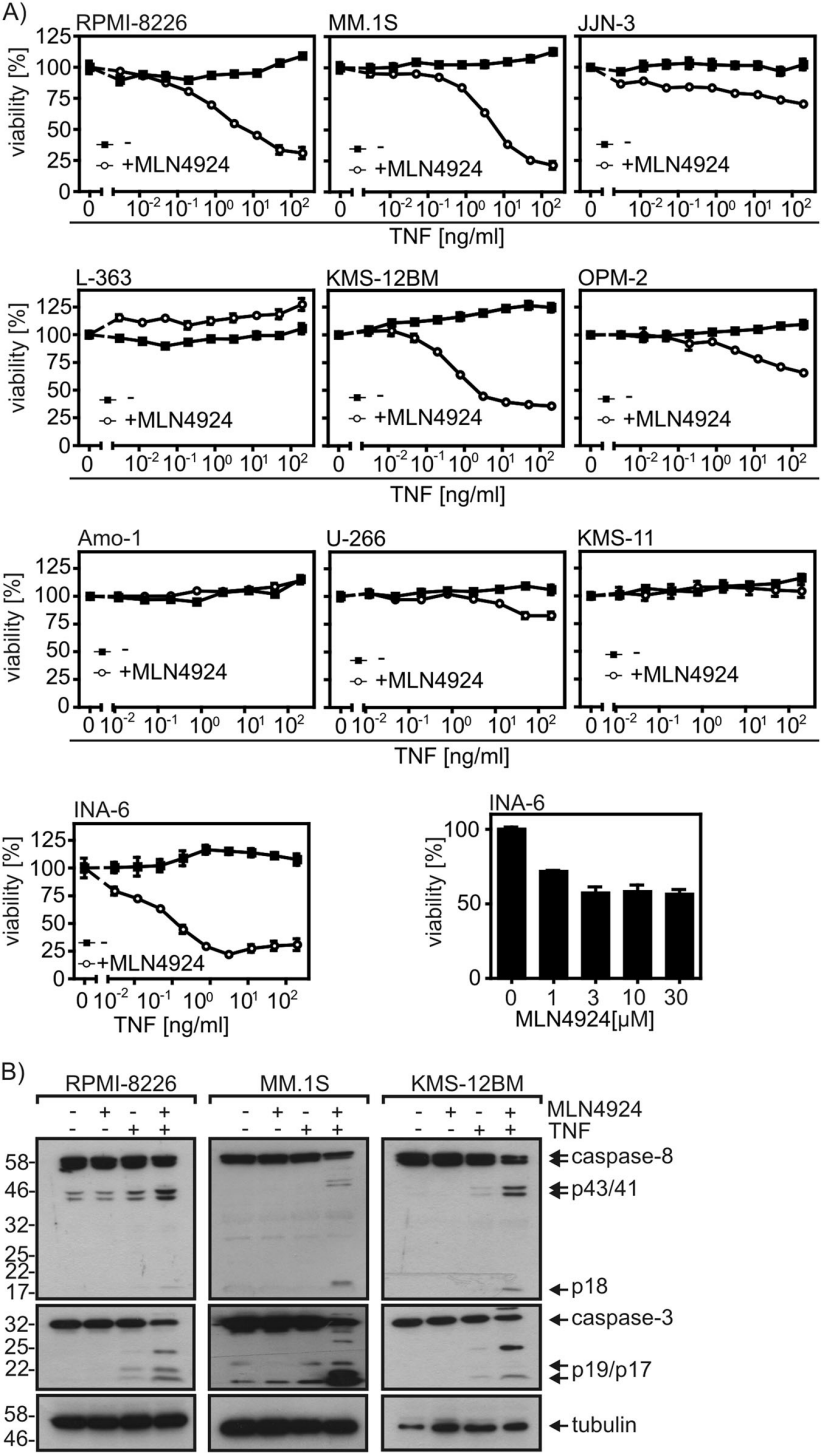
MLN4924 enhances TNF-induced cell death in a subset of myeloma cell lines. **a** Myeloma cell lines were challenged overnight in technical triplicates with the indicated combinations of TNF and MLN4924 (20 µM) and analyzed for cell viability. **b** RPMI-8226, MM.1S and KMS-12BM cells untreated or treated with TNF (100 ng/ml), MLN4924 (20 µM) or a mixture of both for 18 h were analyzed by Western blotting for processing of the indicated proteins. Data shown are representative of at least two independent experiments

The NFκB system has been crucially implicated in the growth and survival of MM cells. The NFκB system is furthermore of overwhelming importance for TNF biology. NFκB signaling not only mediates many of the proinflammatory functions of TNF but also protects most cells from its cell death-inducing activities. Since TNF is typically expressed by immune cells present in the tumor microenvironment of myeloma cells and other cancer entities, we explored the possibility of a synergistic cytotoxic effect of soluble recombinant TNF and MLN4924 on a panel of 10 myeloma cell lines. All multiple myeloma cell lines investigated were resistant against treatment with TNF alone (Fig. 3a). In the presence of MLN4924, however, TNF was strongly cytotoxic on four of the cell lines (RPMI-8226, KMS-12BM, MM.1S, INA-6) and induced minor cell death in three other ones (JJN-3, OPM-2, U-266) (Fig. 3a). Worth mentioning, INA-6 cells were already sensitive to treatment with MLN4924 alone (Fig. 3a, last panel). Cell death induction by TNF and MLN4924 furthermore correlated with synergistic stimulation of processing of apoptotic caspases (Fig. 3b).

**Fig. 4:**
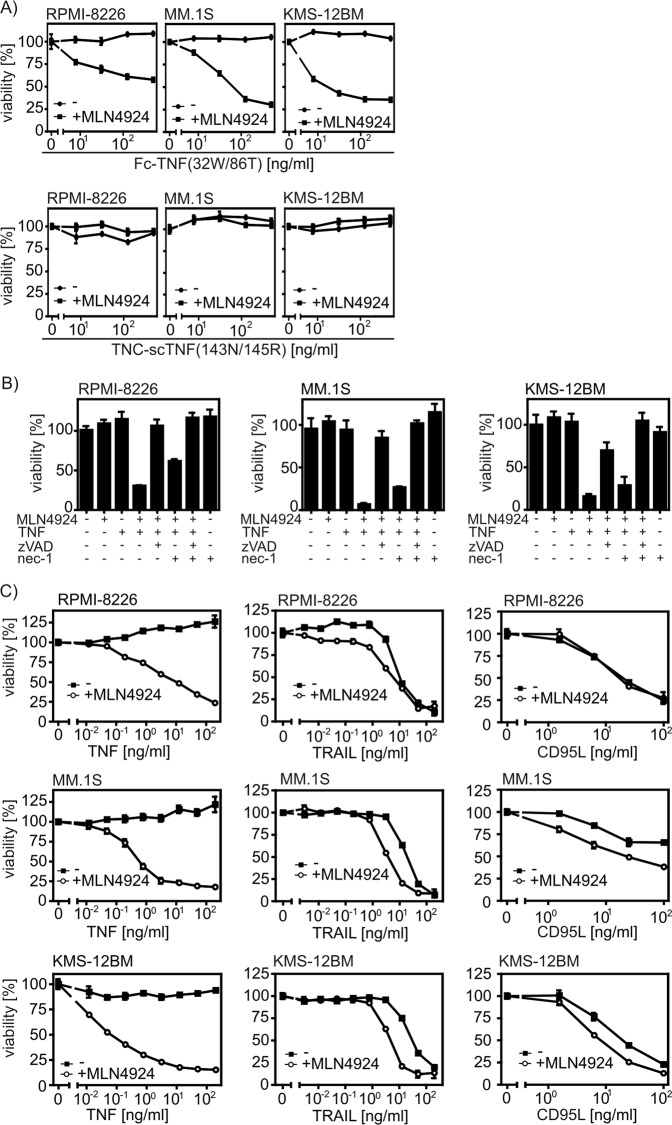
MLN4924 enhances apoptosis and necroptosis induction by TNFR1 in myeloma cells. **a** Cells were stimulated with the TNFR1-specific TNF mutant Fc-TNF(32W/86T) and the TNFR2-specific agonist TNC-scTNF(143N/145R) in the presence and absence of MLN4924 (20 µM). Next day, cells were analyzed for viability. **b** Effect of zVAD-fmk (50 µM) and nec-1 (90 µM) on TNF (100 ng/ml)/MLN4924 (20 µM)-induced cell death after overnight stimulation. **c** Cells were challenged with the indicated combinations of Killer-TRAIL, anti-FLAG mAb M2-oligomerized Flag-CD95L, TNF and 20 µM MLN4924 overnight and were analyzed for cellular viability. Data shown are technical triplicates and representative of at least two independent experiments

TNF interacts with TNFR1 and TNFR2 but only TNFR1 is directly linked to cytotoxic signaling pathways^7^. We found accordingly that only the TNFR1-specific TNF mutant Fc-TNF(32W/86T) but not TNC-scTNF(143N/145R), a highly active TNFR2-specific TNF mutant-based fusion protein^8^, was able to induce cell death in myeloma cells in the presence of MLN4924 (Fig. 4a). Cell death induction by cotreatment of TNF and MLN4924 was blocked in a cell type-dependent manner by the pan-caspase inhibitor zVAD-fmk or a combination of this compound with the RIPK1 inhibitor necrostatin-1 (nec-1) (Fig. 4b) indicating that MLN4924 sensitizes myeloma cell lines for both apoptosis and necroptosis induction by TNFR1. Noteworthy, the cytotoxic activity of TNF-related death ligands TRAIL and CD95L that act by stimulation of the TNFR1 homologous death receptors TRAILR1, TRAILR2, and CD95 remained largely unaffected by MLN4924 (Fig. 4c).

### MLN4924 inhibits TNFR1-induced expression of survival proteins and attenuates formation of the TNFR1-induced signaling complex

**Fig. 5:**
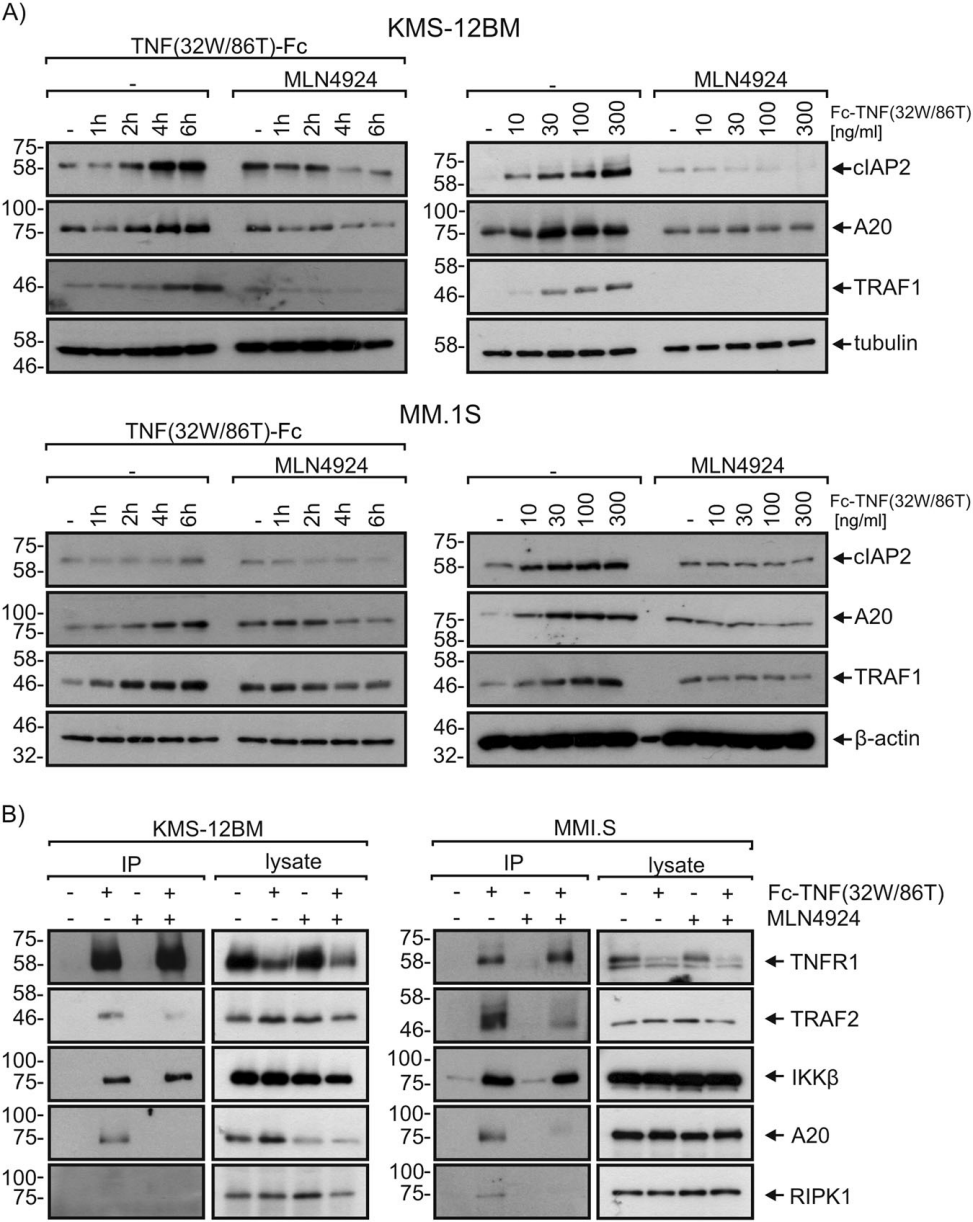
MLN4924 inhibits TNF-induced production of survival proteins. **a** KMS-12BM and MM.1S cells were primed with MLN4924 (20 µM) for 2 h or remained untreated. Cells were then stimulated with 300 ng/ml Fc-TNF(32W/86T) for 1, 2, 4, or 6 h (left panels) or for 6 h with 10, 30, 100, or 300 ng/ml Fc-TNF(32W/86T) (right panels). Total cell lysates were analyzed by Western blotting for the presence of the indicated proteins. **b** Cells were primed with MLN4924 and were then stimulated with Fc-TNF(32W/86T). The TNFR1-associated signaling complex was analyzed by help of protein G immunoprecipitation and Western blotting. Data shown are representative of at least two independent experiments

Deletion of components of the NFκB system in mice frequently results in embryonal death that can be rescued by additional deletion of TNF or TNFR1. The preference of the cell death-sensitizing effect of MLN4924 for TNFR1-induced cell death therefore suggests that this activity of MLN4924 is due to its ability to abrogate NFκB signaling (Fig. 2). In accordance with this idea, we observed that MLN4924 prevented TNF-induced production of the NFκB-regulated factors cIAP2, A20, and TRAF1 (Fig. 5a) which all have been implicated in the inhibition of TNFR1-induced caspase-8 activation^21–24^. In line with the fact that TRAF1 protects TRAF2 from receptor-induced degradation^25^, there was less TRAF2 in the TNFR1 signaling complex of MLN4924-treated cells with reduced TRAF1 induction. Likewise, we noted reduced levels of A20 in the TNFR1 signaling complex of MLN4924-treated cells and also a reduction in the protective ubiquitinated species of RIPK1 (Fig. 5b).

### Only a subgroup of multiple myeloma cell lines expresses TNFR1

**Fig. 6:**
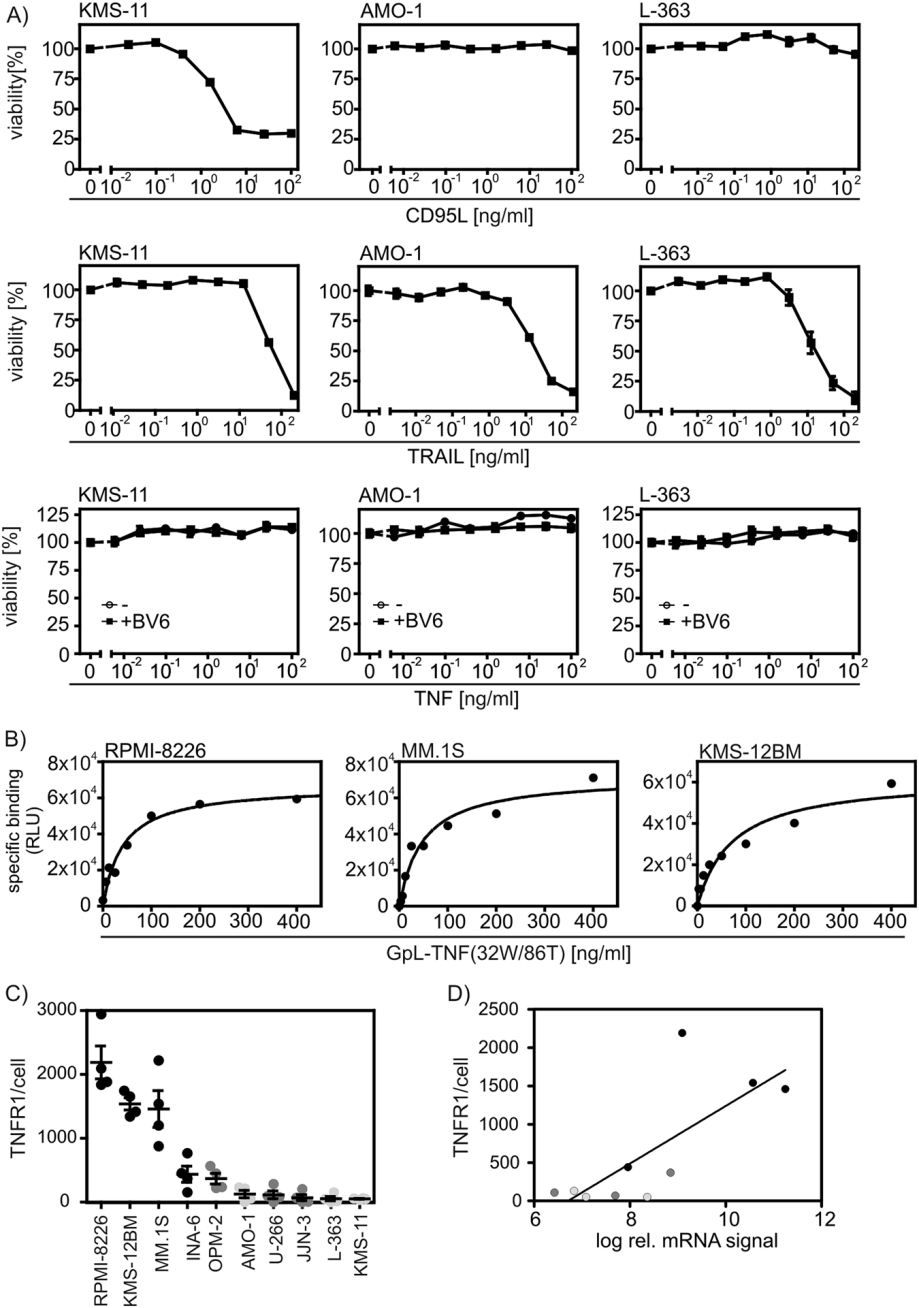
Sensitivity of myeloma cell lines for TNF/MLN4924-induced cell death correlates with TNFR1 expression. **a** Cell lines were challenged overnight with anti-FLAG mAb M2 oligomerized Flag-CD95L, Killer-TRAIL and the indicated combinations of TNF and BV6 (10 µM). Next day, cells were analyzed for cell viability using the MTT assay. Data shown are representative of at least two independent experiments. **b** Affinity of TNC-TNF(32W/86T) for TNFR1 was determined by equilibrium binding studies on ice with RPMI-8226, MM.1S, and KMS-12BM cells. Shown are representative experiments. Average values and statistics of experiments are summarized in Table 2. **c** Absolute cell surface expression of TNFR1 was determined by equilibrium binding studies on ice with 200 ng/ml GpL-TNC-TNF(32W/86T) which ensures >95% receptor occupation. Shown are the results of four independent binding experiments for each cell line. **d** TNFR1 mRNA expression determined by microarray mRNA analysis. See supplementary Table 1 for numerical values

**Fig. 7:**
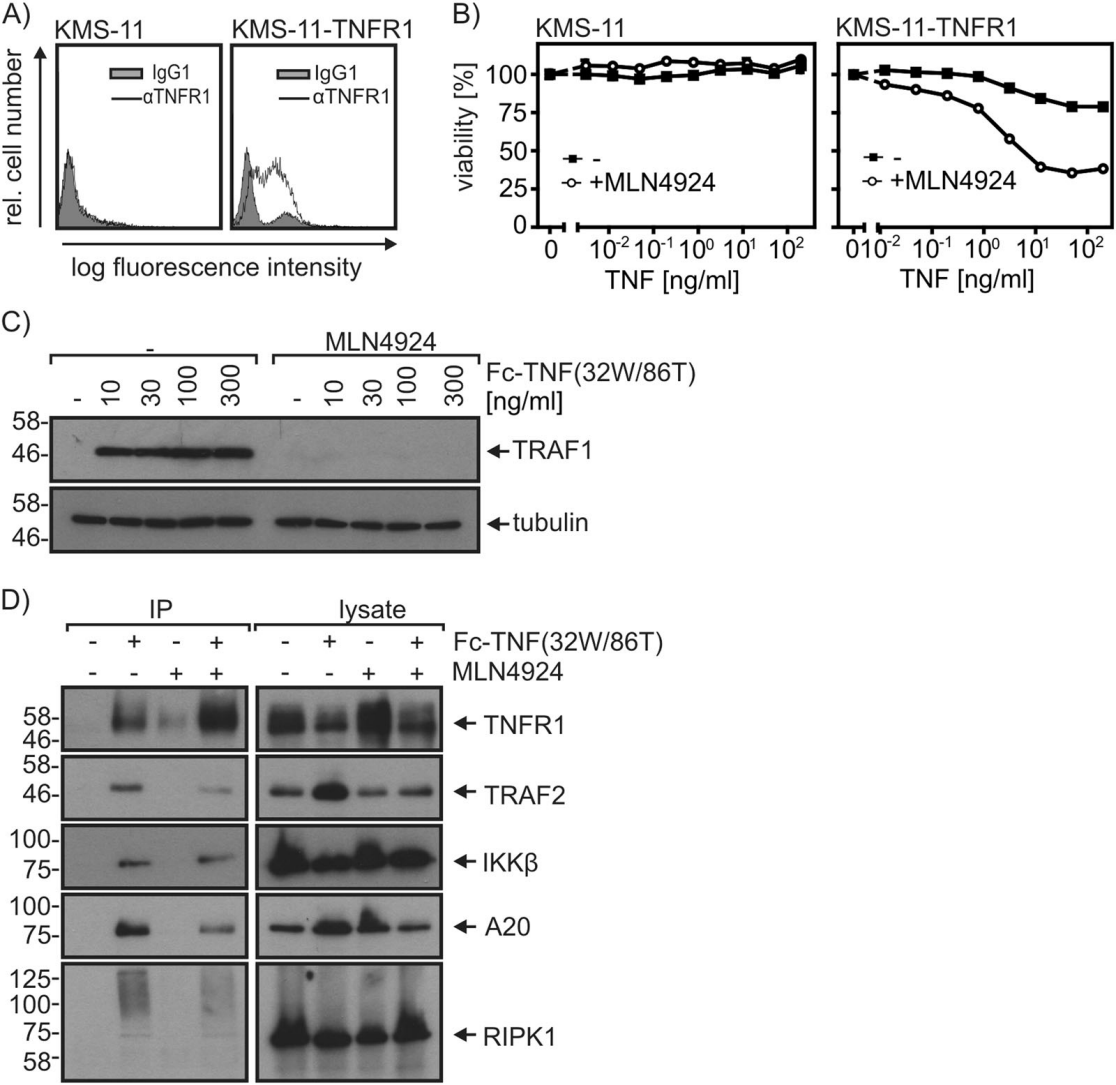
Forced TNFR1 cell surface expression in KMS-11 cells engenders sensitivity for TNF/MLN4924-induced cell death. **a** KMS-11 and KMS-11-TNFR1 cells were analyzed for TNFR1 cell surface expression by FACS. **b** Cells were challenged overnight as indicated in technical triplicates with TNF and MLN4924 (20 µM) and analyzed for cell viability. **c** KMS-11-TNFR1 cells were primed with MLN4924 (20 µM) for 2 h or remained untreated. Cells were then stimulated with the indicated concentrations of Fc-TNF(32W/86T) for 6 h and total cell lysates were analyzed by Western blotting for the presence of the indicated proteins. **d** The TNFR1-associated signaling complex of KMS-11-TNFR1 cells was analyzed after 10 min by protein G immunoprecipitation and Western blotting. Data shown are representative of at least two independent experiments

To figure out the molecular basis of TNF/MLN4924 resistance of myeloma cells, we initially evaluated the sensitivity of the TNF-resistant MM cell lines KMS-11, AMO-1, and L-363 for TRAIL-induced and CD95L-induced cell death. All three cell lines were found to be highly sensitive for CD95L-induced and/or TRAIL-induced cell death (Fig. 6a). Moreover, these cells remained TNF resistant even in the presence of the potent apoptosis sensitizer BV6 (Fig. 6a). These results ruled out that a general defect in death receptor signaling downstream of caspase-8 and RIPK1 is responsible for the observed TNF/MLN4924 resistance. Noteworthy, the sensitivity of the investigated myeloma cell lines for TNF/MLN4924-induced cell death correlated with the relative cell surface expression levels of TNFR1 in these cell lines that we reported in an earlier study^26^. To determine the absolute TNFR1 expression levels, we initially determined the affinity of the GpL fusion protein of TNC-TNF(32W/86T) by equilibrium binding studies with RPMI-8226, MM.1S, and KMS-12BM cells (Fig. 6b). This revealed a KD-value for the TNF-TNFR1 interaction of 42–75 ng/ml (Fig. 6b, Table 2). We then performed binding studies for all cell lines with 200 ng/ml of the GpL-TNC-TNF(32 W/86T) what ensures practically full TNFR1 occupation. This revealed 1460 to 2190 cell surface exposed TNFR1 molecules per cell for the highly TNF/MLN4924-sensitive cell lines RPMI-8226, MM.1S, and KMS-12BM, while the less sensitive INA-6, JJN-3, OPM-2, and U-266 cells expressed only 70 to 437 receptors per cell (Fig. 6c). There was no or very low specific TNFR1 binding in the other insensitive MM cell lines (Fig. 6c). The cell surface exposed expression levels of TNFR1 correlated roughly with TNFR1 mRNA expression (Fig. 6d, supplementary Table I and supplementary fig. S1). There was furthermore no evidence for mutations in the TNFR1 gene in the cell lines investigated here from whole exome sequencing (data not shown). Analysis of the CoMMpass (Relating Clinical Outcomes in MM to Personal Assessment of Genetic Profile) data base of the MMRF Personalized Medicine Initiative (https://research.themmrf.org/) covering 1440 samples furthermore revealed only two mutations in TNFR1, thus far less as for established genes mutated in myeloma, such as TRAF2 and TRAF3 (supplementary Tables II). In sum, our data suggests that the differences in expression levels of TNFR1 in myeloma cell lines is the major factor determining their MLN4924/TNF sensitivity. Indeed, stable transfection of the TNF/MLN4924-resistant KMS-11 cell line with a TNFR1 expression plasmid resulted in a KMS-11 variant undergoing synergistic cell death-induction in response to TNF and MLN4924 (Fig. 7a, b). In this KMS-11-TNFR1 variant, MLN4924 priming again interfered with anti-apoptotic TNFR1 signaling and formation of the non-cytotoxic plasma membrane-associated TNFR1 signaling complex (Fig. 7c, d).

**Table 2:** Determination of the affinity of GpL-TNC-TNF(32W/86T) for TNFR1

Cell line	Number of experiments	single *K*_D_ values (ng/ml)	Averaged *K*_D_ (ng/ml) (mean ± SE)
RPMI-8226	3	46, 137, 41	75 ± 31
MM.1S	3	50, 48, 59	52 ± 3
KMS-12BM	4	73, 41, 15, 37	42 ± 12

### MLN4924 sensitizes a fraction of primary myeloma cells for TNF-induced cell death

**Fig. 8:**
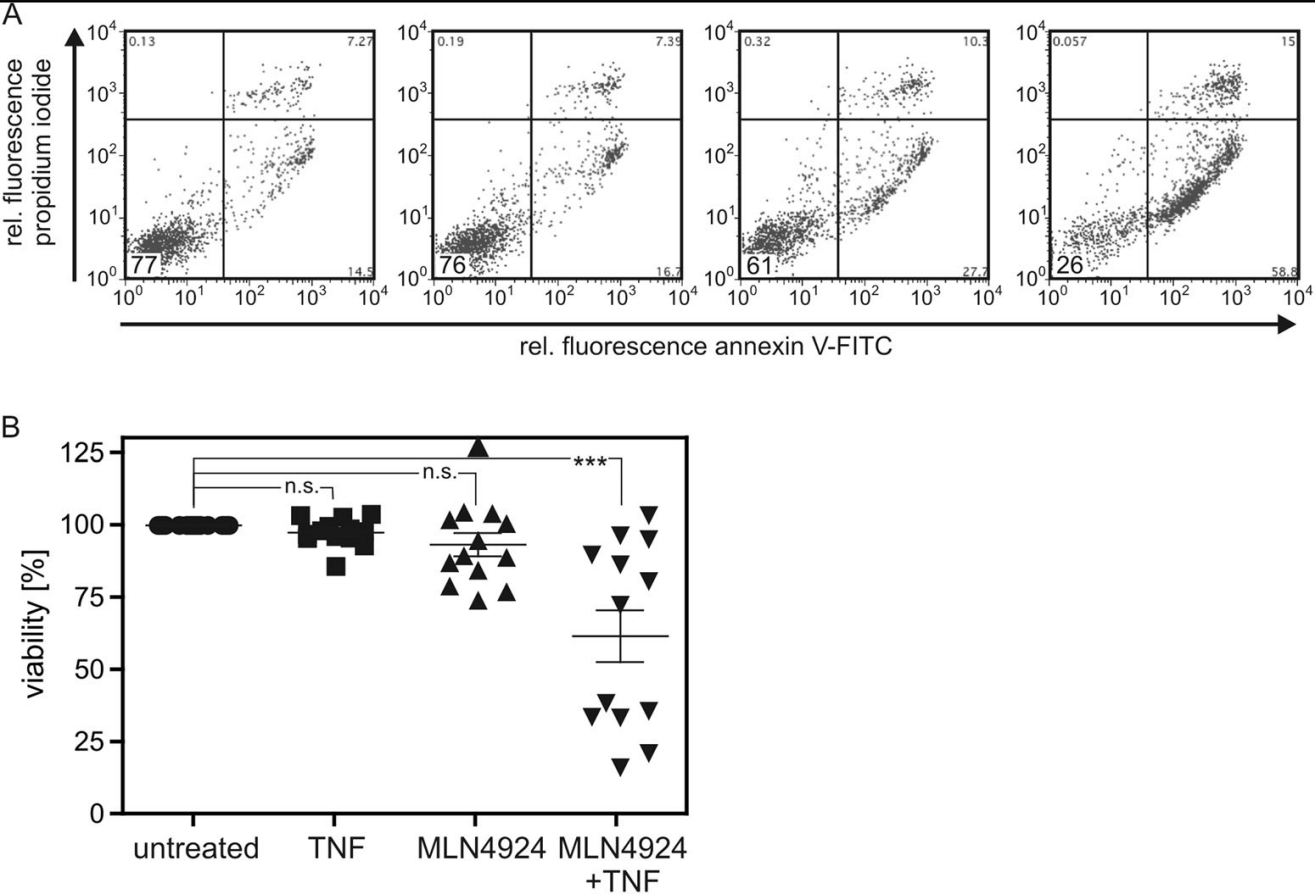
TNF/MLN4924 induces apoptosis in primary myeloma cells of a subgroup of patients. **a**, **b** Freshly purified primary MM cells were incubated with either TNF (100 ng/ml), MLN4924 (20 µM), a combination thereof or 1:1000 DMSO (the solvent for MLN4924). After 1 day annexin V/propidium iodide (PI) staining was performed by flow cytometry. A representative annexin V/propidium iodide (PI) staining panel is shown for one sample (# 11). % cells in the double-negative quadrant are highlighted. **a** Annexin V/PI double-negative cells (lower left quadrant) were considered as the “live cell fraction”. Relative survival, as shown in **b**, was obtained by normalization of the annexin V/PI double-negative fraction of each treatment group to the corresponding live cell fraction observed in the DMSO-treated control group. Results were analyzed by ANOVA (one-way, Bonferroni comparison of all pairs of columns. ***p < 0.001; n.s. not significant. Please note, considering TNF + MLN4924 treated cells showing >50 and <50% viability as separate groups resulted in again in significant (***p < 0.00) killing for the “<50%” subgroup and no significant difference for the “>50% subgroup

To further substantiate the relevance of our findings, we also investigated CD138^+^ cells isolated from bone marrow aspirates of 12 MM patients (Table 3). For one patient two samples, one before and one after treatment, were available. After isolation, the primary myeloma cells were stimulated with TNF and/or MLN4924. The next day, cells were analyzed by annexin V/propidiumiodide (PI) staining and flow cytometric analysis. Absolute survival, thus the fraction of double-negative cells, ranged between 36 and 82% and was 60% in average (supplementary data III). The relative survival of cells treated with TNF, MLN4924, or TNF + MLN4924 was defined for each primary cell sample as the ratio of the double-negative cells observed in these groups and the double-negative cells obtained in the DMSO-treated control group. Relative survival of TNF-treated myeloma cells was between 86% and 105% (Fig. 8b). There was no statistical significant difference in cell survival between the TNF-treated and DMSO-treated control group. Relative survival of MLN4924-treated myeloma cell was somewhat more variable and reached from 74% to 126% (Fig. 8b). Nevertheless, there was also no significant difference in cell survival in comparison to the DMSO-treated control group. In 6 of the 13 samples, however, there was synergistic killing by TNF and MLN4924 reducing relative survival to 17–39% and reaching significance for the whole TNF + MLN4924-treated panel (Fig. 8b). Please note, when TNF + MLN4924 treated cells with >50% relative viability were analyzed as a separate group this resulted in no significant difference to the control group. Thus, the sensitivity for TNF + MLN4924 treatment defines two subgroups of primary MM cells and mirrored in this regard the situation obtained with the human myeloma cell line panel. It is therefore tempting to speculate that cell surface expression of TNFR1 not only decides whether myeloma cell lines are sensitive for TNF-MLN4924 mixtures but also determines the responsiveness of primary myeloma cells for this drug combination. The proof of correlation of TNFR1 cell surface expression of primary myeloma cells with their TNF/MLN4924 sensitivity was not possible because standard FACS analysis of TNFR1 expression is too insensitive to detect functionally relevant amounts of this receptor, while more sensitive binding studies with iodinated or luciferase labeled TNFR1-specific antibodies or TNFR1-specific TNF mutants were not feasible with the limited quantities of primary MM cells available.

**Table 3:** Primary samples derived of myeloma patients

Patient#	Sample#	Age	Gender	Ig subtype	Stage	Therapies	FISH
1	1	64	f	IgG lambda	III A	None	Tri9q
	2	64	f	IgG lambda	III A	3× RAD^a^	Tri9q
2	3	50	m	IgG kappa	III A	Dexa^b^ 4× RAD 2× HD-Mel^c^ Len^d^	Normal
3	4	73	m	IgG kappa	I A	None	Hyperdiploide CMYC-Rearrangement
4	5	58	f	IgG lambda	I A	None	Amp1q21
5	6	78	f	IgG lambda	II A	None	–
6	7	72	m	IgG kappa	I A	None	–
7	8	43	m	IgA kappa	III A	None	Hyperdiploid
8	9	68	f	IgG kappa	III A	None	Hyperdiploid
9	10	49	f	IgG kappa	III A	None	Tri9q
10	11	61	m	Kappa LC	III B	3× ID > 2× HD-Mel; VRCD^e^ > HD-Mel; VRCD	Normal
11	12	67	m	IgG lambda	II A	None	Hyperdiploid
12	13	62	m	Kappa LC	II A	None	Tri1q

^a^radiation

^b^dexamethason

^c^high-dose melphalan

^d^lenalodimide

^e^bortezomib dexamethasone thalidomide cyclophosphamide

## Discussion

TNF is an extremely pleiotropic cytokine which exerts its cellular effects by stimulation of two receptors of the TNF receptor (TNFR) superfamily (TNFRSF): TNFR1 and TNFR2^27^. TNF stimulates a broad range of cell responses dependent on cell type and cellular context. This includes such opposing effects as transcriptional upregulation of proinflammatory and cell protective proteins but also the activation of apoptotic and necroptotic cell death^27^. The two receptors of TNF belong to two distinct subgroups of the TNFRSF. TNFR1 is a death receptor (DR) and thus possesses a death domain (DD) in its cytoplasmic part which allows recruitment of defined signaling proteins also carrying a DD^28^. These DD-containing signaling proteins connect TNFR1 with cytotoxic signaling pathways but also with stimulation of the IKK complex and activation of the classical NFκB pathway. Importantly, the cytotoxic signaling potential of TNFR1 is typically suppressed by varying mechanisms including constitutive and NFκB-dependent expression of anti-apoptotic proteins and modification of signaling proteins involved in cytotoxic TNFR1 signaling, such as RIPK1^27–29^. The default mode of TNFR1 signaling is therefore typically proinflammatory. TNFR2 is a TNF receptor associated factor (TRAF)-interacting TNFRSF receptor. As such, TNFR2 has no DD but is able to recruit the adapter protein TRAF2 along with its interacting proteins TRAF1 and cellular inhibitor of apoptosis protein-1 (cIAP1) and cIAP2^28^. TNFR2 has thus no own authentic cell death stimulating activity. Noteworthy, TRAF1, TRAF2, cIAP1, and cIAP2 are also indirectly recruited to TNFR1 by the DD-containing signaling proteins TRADD and RIPK1 and fulfill there a dual function. TRAF1/2 and the cIAPs not only promote proinflammatory TNFR1 signaling but also suppress its cytotoxic signaling abilities^27,29^.

While TNFR1 is ubiquitously expressed, TNFR2 expression is limited to certain cell types, particularly myeloid cells and a fraction of T cells and B cells^30^. TNF is a highly inducible cytokine which is produced by activated immune cells, especially macrophages and T cells^31,32^. TNF expression can also be induced in non-immune cells, e.g., fibroblasts, endothelial cells, and epithelia cells by various proinflammatory proteins including TNF itself^33–35^. In general the tumor microenvironment contains a high frequency of potentially TNF producing cells and resembles in many aspects chronic inflamed tissues^36^. Thus, tumor cells are frequently exposed to TNF.

MLN4924 inhibits the NEDD8 activating enzyme and prevents so the degradation of the CRL substrate phospho-IκBα which is generated by the IKK complex^14,15^. MLN4924 therefore acts as an inhibitor of TNF-induced NFκB activation downstream of the activation of the IKK complex. In accordance with the fact that TNFR1-induced NFκB signaling contributes to cell death resistance against TNF, we and others reported that MLN4924 can sensitize cells for the cytotoxic activities of TNF^19,20^. MLN4924 is currently under consideration in a variety of clinical trials including phase 1 studies with patients suffering on acute myeloid leukemia, myelodysplastic syndromes, metastatic melanoma, relapsed/refractory MM or lymphoma and solid cancer^37–42^. Since TNF has been identified as a factor which is expressed in MM^43–45^, we evaluated in this study the different multiple myeloma cell lines and primary myeloma cell samples with respect to their TNF sensitivity in the presence of MLN4924. A subset of multiple myeloma cell lines and also about half of the primary MM samples were found to be sensitized for TNF-induced cell death by MLN4924 (Figs. 3 and 8). Not unexpectedly, selective TNFR1 stimulation was fully sufficient to trigger cell death (Fig. 4) despite the often high expression levels of TNFR2 in myeloma cells^26^. Interestingly, MLN4924 treatment showed no or only a moderate sensitizing effect on the TNFR1-related death receptors TRAILR1/2 and CD95 (Fig. 4c). Apoptotic signaling by these DRs and TNFR1 merge at the level of FADD-mediated caspase-8 activation^40^. The TNFR1-selective activity of MLN4924 suggests therefore that MLN4924 affects cell death survival mechanisms upstream of FADD and caspase-8 in TNFR1 signaling. NFκB signaling bifurcates from cell death signaling upstream of FADD in case of TNFR1 but downstream of FADD in case of CD95 and TRAIL death receptors^40^. Indeed, in the presence of MLN4924 TNF-induced activation of the classical NFκB pathway was strongly inhibited (Fig. 2a). Some of the NFκB-regulated factors such as TRAF1, A20, and cIAP1/2 are recruited into the TNFR1 signaling complex to antagonize the pro-cell death activities of RIPK1^27–29^. In accordance with this, we observed in MLN4924 treated cells reduced RIPK1 modification and reduced recruitment of TRAF2 (Fig. 5).

A striking observation was that only a subset of the myeloma cell lines and about half of the primary MM samples were killed by TNF/MLN4924. We found earlier that some MM cell lines express no or functionally negligible levels of TNFR1^26^, and therefore investigated to which extent TNF/MLN4924 sensitivity and TNFR1 expression correlate in MM. Highly sensitive and accurate binding studies with a *Gaussia princeps* luciferase fusion protein of a TNFR1-specific TNF mutant and qPCR analysis revealed that TNFR1 expression in myeloma cell lines correlated with high TNF/MLN4924 sensitivity (Fig. 6). In line with this, TNFR1 expression in a MM cell line largely lacking endogenous TNFR1 expression was fully sufficient to restore TNF/MLN4924 sensitivity (Fig. 7). Thus, MLN4924 might be particularly efficient in the treatment of MM patients with high TNFR1 expression on MM cells and a TNF^high^ tumor microenvironment. In this scenario, the effect of MLN4924 on the interplay of tumor-associated TNF and cells of the tumor microenvironment is presumably also of relevance. Since MLN4924 inhibits TNF-induced activation of the classical NFκB pathway, it has the potential to prevent protumoral effects of TNF resulting from TNFs ability to induce the production of cytokines and growth factors in non-transformed cells. Indeed, we observed in initial experiments that MLN4924 inhibits TNF-induced but also basal IL-6 production by bone marrow-derived stroma cells. The sensitizing effect of MLN4924 for TNF-induced cell death might also affect cells of the tumor microenvironment, e.g., tumor associated T cells and/or myeloid cells. The consequences for the tumor resulting from TNF-induced killing of these cell types are, however, complex and yet not predictable.

## Materials and methods

### Cell lines and reagents

The cell lines KMS-12BM, AMO-1, JJN-3, L-363, OPM-2, RPMI-8226, and U266 were from the German Collection of Microorganisms and Cell Cultures (DSMZ, Braunschweig, Germany), MM.1S cells were from LGC Standards (Wesel, Germany) and the INA-6 cell line were a kind gift from Martin Gramatzki (University Medical Center Schleswig-Holstein, Kiel, Germany). MM cell lines were cultured in RPMI 1640 medium supplemented with 10% fetal bovine serum. INA-6 cells were cultured in the presence of 2 ng/ml IL-6. The antibodies used in this study were purchased from Cell Signaling Technology, Beverly, MA, USA (anti-caspase-3, #9662; anti-caspase-9, #9502; anti-cellular inhibitor of apoptosis protein 2 (cIAP2), #3130; anti-β-catenin, clone 6B3; anti-IκBα, clone L35A5; anti-phospho-IκBα, clone 14D4; anti-p100/p52, #4882; anti-A20, #5630; anti-TRAF1, #4715; anti-TRAF2, #4724; anti-TNFR1, #3736; anti-IKKβ, clone D30C6; anti-NEDD8, clone 19E3); Enzo Life Sciences, Lörrach, Germany (anti-caspase-8, clone C15); BD Biosciences, Germany (anti-RIPK1, clone 38/RIPK1); Dunn Labortechnik, Asbach, Germany (anti-tubulin-α, clone DM1A); R&D Systems, USA (PE-labeled anti-TNFR1, clone # 16803; anti-IgG1 (mouse IgG1, clone 11711); Sigma-Aldrich, St. Louis, MO, USA (anti-β-actin, clone AC-15) and Santa Cruz Biotechnology, Germany (anti-Nrf2, clone H-300). zVAD-fmk was obtained from Bachem AG, Weil am Rhein, Germany and nec-1 was obtained from Enzo Life Sciences. Human TNF-α was a kind gift from Prof. Daniela Männel (University of Regensburg, Germany). GpL-TNC-TNF (32W/86T), Fc-TNF(32W/86T) (TNFR1-specific TNF mutant), TNC-scTNF(143N/145R) (TNFR2-specific nonameric TNF mutant)^8^ and Flag-CD95L were produced in HEK293 cells. Killer-TRAIL was purchased from Enzo Life Sciences. MLN4924 was obtained from Active Biochemicals Co., Hong Kong, China, carfilzomib was obtained from Selleck Chemicals (Munich, Germany) and bortezomib from LC Laboratories, Woburn, MA, USA. The SMAC mimetic BV6 was obtained from Syngene (Bangalore, India).

### Western blotting

Cells were harvested in ice-cold PBS, were washed twice in PBS, pelleted by centrifugation (4 min, 1200 rpm, 4 °C, Mikro 200 R, Eppendorf rotor) and finally total cell lysates were prepared by sonicating cells for 20 s in 4× Laemmli buffer (0.2 M Tris, 10% β-mercaptoethanol, 8% SDS, 40% glycerol, pH 8.0) supplemented with phosphatase inhibitor mixture II (Sigma) and boiling at 95 °C for 5 min. Cell lysates were subjected to vertical SDS-PAGE, and proteins were transferred to nitrocellulose membranes by wet blotting. Blocked membranes were incubated overnight at 4 °C with the primary antibody of interest and the antigen-primary antibody complexes were detected using anti-mouse-HRP (Dako-Cytomation, Hamburg, Germany) or anti-rabbit-HRP (Dako-Cytomation or Cell Signaling Technology) and the ECL Western blotting detection kit and analysis system from Thermo Fisher Scientific (Darmstadt, Germany).

### Evaluation of cellular viability

Cells were seeded in 96-well tissue culture plates (60 × 10^3^ per well). Cells were treated in triplicates with the indicated stimuli and cell viability was determined the next day using the 3-(4,5-dimethylthiazol-2-yl)-2,5-diphenyltetrazolium bromide (MTT) staining method. In all experiments investigating MLN4924, the compound was added 30 min before cells were treated with TNF etc. Flag-CD95L was oligomerized by incubating the ligand with FLAG-specific mAb M2 (1 µg/ml, Sigma-Aldrich, St. Louis, MO, USA) 30 min before cell stimulation. In experiments using zVAD-fmk/nec-1, cells were treated with zVAD-fmk (50 µM), nec-1 (90 µM) or zVAD-fmk/nec-1 30 min before stimulation with TNF etc. Cell viability values were normalized according to cells without TNF treatment (defined as 100% viable) and cells that have been incubated with a highly cytotoxic cocktail containing sodium azide (defined as 0% viable).

### Determination of the TNFR1 affinity of GpL-TNC-TNF(32W/86T)

Equal aliquots of cells were incubated with conventional TNF (50 µg/ml) for 1 h on ice or left untreated. Cells were then incubated pairwise for another hour with increasing concentrations of GpL-TNC-TNF(32W/86T). Then, cells were washed three times with ice cold PBS to remove unbound GpL-TNC-TNF(32W/86T). Cell pellets were finally suspended in 50 µl RPMI culture medium with 0.5% FBS, transferred to a black 96-well plate and assayed for luciferase activity with the Luciferase Assay Kit from New England Biolabs GmbH using a Lumo laminator (Autobio-Diagnostics). Luciferase activities of GpL-TNC-TNF(32W/86T)-treated cells were considered as total binding and luciferase activities of TNF-pretreated/GpL-TNC-TNF(32W/86T)-treated cells were considered as nonspecific binding. Specific binding values were calculated as the difference between total and non-specific binding values and analyzed by nonlinear regression to a one-site specific binding curve with the GraphPad Prism 5 software to obtain the *K*_D_-value of the interaction of TNFR1 with GpL-TNC-TNF(32W/86T).

### Determination of the average number of TNFR1 molecules per cell

Cells (5 × 10^5^) were incubated for 1 h on ice with 200 ng/ml GpL-TNC-TNF(32W/86T) with and without preincubation with TNF (50 µg/ml) for 1 h on ice. Then, cells were washed three times and the experiment was proceeded further as described in the previous section. Cells were counted at the end of each experiment to calculate by help of the measured specific GpL activity of the TNC-GpL-TNF(32W/86T) preparation and the cell bound specific GpL activity, the number of TNFR1 molecules per cell.

### Analysis of mRNA expression

The expression of TNFR1 mRNA in MM cell lines was determined using HG-U133 plus 2.0 microarrays from Affymetrix (Affymetrix, Santa Clara, CA, USA). Gene expression data was normalized using the MAS5 algorithm and subsequently log2 transformed and annotated to gene symbols using the madb database (https://madb.nci.nih.gov/index.shtml).

### Immunoprecipitation

Cells were seeded in cell culture petri dishes, were treated with MLN4924 (20 µM) overnight and stimulated the next day with Fc-TNF(32W/86T) (2 µg/ml) for 10 min. Cells were harvested and washed twice with ice-cold PBS and lysed for 30 min on ice in 30 mM Tris-HCl (pH 7.5), 1% Triton X-100, 10% glycerol, 120 mM NaCl supplemented with a protease inhibitor (Roche Diagnostics, Germany) cocktail. Lysates were cleared by centrifugation (1st centrifugation: 5000 rpm, 4 °C, 5 min; 2nd and 3rd centrifugations: 14000 rpm, 4 °C, 20 min, Mikro 200R, Eppendorf rotor) and Fc-TNF (32W/86T)-associated TNFR1 complexes were precipitated from the cleared lysates using protein G-agarose beads (30 μl 50% slurry) at 4 °C overnight. Lysates of untreated and MLN4924 alone groups were supplemented with 5 ng Fc-TNF(32W/86T) to serve as a negative control. The next day, protein G beads were washed five times with ice cold lysis puffer and beads were incubated in Laemmli buffer for 15 min at 85 °C to release bound proteins. Protein samples were fractionated and analyzed as described in the Western blotting section.

### Generation of KMS-11 cells with stable TNFR1 expression

The coding sequence of human TNFR1 was amplified and flanked with NheI and NotI sites using primers 5′-GCTGGCTAGCATGGGCCTCTCCACCGTGCC and 5′-CAGGGCGGCCGCCTCATCTGAGAAGACTGGGCG, and cloned into a CMV promotor-based expression cassette of a puromycin resistance-mediating *Sleeping Beauty* vector. After electroporation into KMS-11 cells (for details see ref. ^46^) a polyclonal stably transposed culture was established after about two weeks of puromycin selection and TNFR1 cell surface expression assessed. For this, KMS-11 and KMS-11-TNFR1 cells were washed with PBS and incubated with PE-labeled anti-TNFR1 or the corresponding isotype control IgG1 for 30 min at 4 °C. Afterwards, cells were washed three times with ice cold PBS and were then analyzed using a FACSCalibur (BD Biosciences, Heidelberg, Germany).

### Ethics statement and purification of primary MM cells

Bone marrow aspirates from MM patients were obtained at the University Hospital of Würzburg, Department of Internal Medicine II, within the frame of diagnostically indicated aspirations and after informed written consent of the respective patients. Permission was granted by the Ethics Committee of the Medical Faculty, University of Würzburg, Würzburg, Germany (reference numbers 18/09, 76/13). The purification of primary MM cells via microbead selection of CD138-positive cells is described in detail in ref. ^47^. Primary cells were not supplemented with IL-6.

### Mass spectrometry

For a detailed description please see supplementary methods in supplementary data.

## Supplementary information

supplemental data
