# Supplementary material for: The NEDD8-activating enzyme inhibitor MLN4924 sensitizes a TNFR1+ subgroup of multiple myeloma cells for TNF-induced cell death
Source: Cell Death Dis. 2019 Aug 13;10(8):611. doi: 10.1038/s41419-019-1860-2 (PMC6690881; doi:10.1038/s41419-019-1860-2)
Supplement: Supplementary file 1 — supplemental data [file 41419_2019_1860_MOESM1_ESM.pdf]

SUPPLEMENTAL DATA

Supplemental figure 1

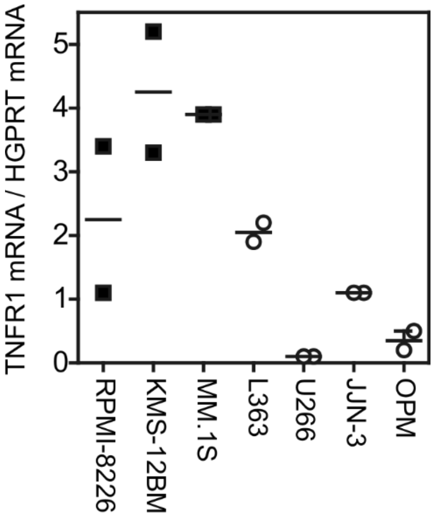

Two independently obtained total RNA samples of each of the three MM cell lines with high TNFR1 mRNA levels from the microarray mRNA analysis shown in figure 6D (filled squares, RPMI-8226, KMS-12BM and MM.1S) as well as two independent samples for each of four cell lines with low TNFR1 expression (white circles, L363, U266 JJN3 and OPM) were analyzed by qRT-PCR for TNFR1 expression.

Total RNAs were isolated with the RNeasy mini kit (Quiagen, Valencia, CA, USA) according to manufacturer's instructions. Two micrograms of total RNA were transcribed into complementary DNA using the high-capacity cDNA reverse transcription kit (Applied Biosystems, Carlsbad, CA, USA). Tnfrsf1a mRNA levels were quantified using the TaqMan human Tnfrsf1a (Hs01042313_m1) gene expression assay (Applied Biosystems) and an ABI Prism 7900 sequence detector (Applied Biosystems). qRT-PCR reactions were performed in quadruplicates for each sample and were normalized to the expression oft he housekeeping gene Hprt1 (Hs02800695_m1). mRNA levels were calculated using the SDS 2.1 software (Applied Biosystems).

Supplemental table I

Expression of TNFR1 and GAPDH mRNA in MM cell lines determined by microarray analysis. For comparison absolute TNFR1 number derived from binding studies of figure 6 were included.

**Cell line**	**Log 2 mRNA signal**		**TNFR1/cell**
	**TNFR1**	**GAPDH**	
**JJN3**	7,693583	15,441320	70
**AMO1**	6,835048	15,170820	130
**INA6**	7,958666	14,924380	440
**KMS11**	8,362409	14,794290	50
**KMS12B**	11,244920	14,777420	1460
**L363**	7,083690	15,613360	50
**MM1.S**	10,568790	15,579100	1540
**OPM**	8,848711	15,614340	370
**RPMI**	9,090881	14,772400	2190
**U266**	6,426434	15,230370	110

Supplemental table II

Mutations in the TNFRSF1A, TRAF2 and TRAF3 genes in the CoMMpass (Relating Clinical Outcomes in MM to Personal Assessment of Genetic Profile) data base with 1440 patients.

Gene	Patient	Mutation	Change Type	Frequence (%)
TNFR1	MMRF1434	T358M	missense variant	0,14
	MMRF2068	T411M	missense variant	
TRAF2	MMRF1180	p.Q67*	stop gained	1,25
	MMRF1180	p.T186fs	frameshift variant	
	MMRF1586	p.E531*	stop gained	
	MMRF1602	D93H	missense variant	
	MMRF1625	p.S411*	stop gained	
	MMRF1801	D93N	missense variant	
	MMRF1822	9:139794125 G>A	splice	
	MMRF1917	p.E210*	stop gained	
	MMRF1965	F433L	missense variant	
	MMRF1999	S89L	missense variant & splice	
	MMRF2041	p.Q509*	stop gained	
	MMRF2166	p.Q67*	stop gained	
	MMRF2166	p.E79*	stop gained	
	MMRF2166	E101K	missense variant	
	MMRF2201	C112S	missense variant	
	MMRF2201	T113A	missense variant	
	MMRF2307	R42L	missense variant	
	MMRF2341	p.E187*	stop gained	
TRAF3	MMRF1078	D463N	missense variant	4,9
	MMRF1078	D552H	missense variant	
	MMRF1128	C76S	missense variant	
	MMRF1252	p.E41fs	frameshift variant	
	MMRF1261	p.A327fs	frameshift variant	
	MMRF1285	p.E174*	stop gained	
	MMRF1327	p.K52fs	frameshift variant	
	MMRF1388	p.Q294*	stop gained	
	MMRF1413	H136L	missense variant	
	MMRF1413	p.L137*	stop gained	
	MMRF1497	Y51fs	frameshift variant	
	MMRF1506	G462W	missense variant	
	MMRF1534	Y39C	missense variant	
	MMRF1572	L543Q	missense variant	
	MMRF1605	p.R341fs	frameshift variant	
	MMRF1668	T469K	missense variant	
	MMRF1672	p.T46fs	frameshift variant	
	MMRF1705	G462R	missense variant	
	MMRF1723	D552Y	missense variant	
	MMRF1725	p.S85fs	frameshift variant	
	MMRF1749	S563W	missense variant	
	MMRF1749	p.D564fs	frameshift variant	
	MMRF1778	p.L496fs	frameshift variant	
	MMRF1778	p.Q342*	stop gained	
	MMRF1785	I477F	missense variant	
	MMRF1785	p.Q492*	stop gained	
	MMRF1796	p.E346*	stop gained	
	MMRF1796	D348H	missense variant	
	MMRF1796	D510N	missense variant	
	MMRF1810	p.K429*	stop gained	
	MMRF1823	W420R	missense variant	
	MMRF1823	L473P	missense variant	
	MMRF1823	F474V	missense variant	

MMRF1833	p.Q407*	stop gained
MMRF1891	S442R	missense variant
MMRF1891	p.Y482*	stop gained
MMRF1908	p.C177fs	frameshift variant
MMRF1911	14:103338307 T>A	splice
MMRF1967	p.Q14*	stop gained
MMRF1999	F445L	missense variant
MMRF1999	D483N	missense variant
MMRF2041	p.S84*	stop gained
MMRF2041	p.R310*	stop gained
MMRF2058	p.R321*	stop gained & splice
MMRF2097	Y548N	missense variant
MMRF2097	p.Y548*	stop gained
MMRF2118	C88W	missense variant
MMRF2118	R428Q	missense variant
MMRF2132	p.W344*	stop gained
MMRF2144	p.Y39fs	frameshift variant
MMRF2151	V494G	missense variant
MMRF2166	14:103336521 G>A	splice
MMRF2170	E313fs	frameshift variant
MMRF2197	p.W488*	stop gained
MMRF2209	p.R310*	stop gained
MMRF2211	p.G416fs	frameshift variant
MMRF2213	14:103338307 T>G	splice
MMRF2224	p.E54*	stop gained
MMRF2265	p.Thr553 Ile556del	inframe deletion
MMRF2271	p.R163*	stop gained
MMRF2271	p.M497fs	frameshift variant
MMRF2352	I323L	missense variant
MMRF2352	p.Q326fs	frameshift variant
MMRF2352	p.Q501*	stop gained
MMRF2377	D98G	missense variant
MMRF2428	L418P	missense variant
MMRF2457	V59E	missense variant
MMRF2515	W488C	missense variant
MMRF2525	p.Y452fs	frameshift variant
MMRF2601	D463N	missense variant

Supplemental table III

Flow cytometry results (percent cells in each quadrant of AV/PI staining) of all primary MM samples and treatments. Quadrant 4 results showing double-negative viable cells are highlighted with a grey background.

Sample #	DMSO				TNF				MLN				TNF + MLN			
	**Q1 Ax- PI+**	**Q2 Ax+ PI+**	**Q3 Ax+ PI-**	**Q4 Ax- PI-**	**Q1 Ax- PI+**	**Q2 Ax+ PI+**	**Q3 Ax+ PI-**	**Q4 Ax- PI-**	**Q1 Ax- PI+**	**Q2 Ax+ PI+**	**Q3 Ax+ PI-**	**Q4 Ax- PI-**	**Q1 Ax- PI+**	**Q2 Ax+ PI+**	**Q3 Ax+ PI-**	**Q4 Ax- PI-**
1	2,1	14,6	16,3	67	2,5	13,6	14,4	69,6	2,5	13,7	13,7	70,1	3,4	17,3	19,1	60,2
2	0,9	34,3	23,3	41,5	1,3	31,2	27	40,6	1,5	29	37,4	32,1	0,4	31,2	59,4	9
3	0,3	11,2	8,3	80,2	0,3	13,6	11,6	74,6	0,3	10,1	7,8	81,8	0,1	41,2	45,3	13,5
4	0,8	22,2	12,9	64,1	0,3	17,7	15,7	66,3	0,5	21,8	20,5	57,1	0,3	17,7	15,7	66,3
5	1,5	42,9	5,6	51	2,1	42,7	5,7	49,5	1,5	47,3	6,7	44,5	0,9	50,2	7,7	41,2
6	0,9	31,8	5,1	62,2	0,3	33,5	4,7	61,5	0,9	31,1	4,2	64,8	0,4	34,5	5,1	60
7	0,6	16,4	47,5	35,6	0,3	17,7	45,4	36,6	0,1	16	38,7	45,2	0,1	33,9	53,1	12,9
8	3,3	13,2	43,2	40,3	2	13,5	44,3	40,2	2,9	17,3	45,6	34,1	0,7	17,7	65,9	15,7
9	0,3	15,5	12,1	72,1	0,2	18	12,8	69	0	19,8	15,6	64,6	0,1	20,5	17,1	62,4
10	1,7	38,4	3,7	56,2	1,6	39,9	4,4	54,1	1,3	37,6	4,5	56,6	0,7	49,4	9	40,8
11	0,3	7,2	15,1	77,4	0,1	7	16,9	76	0,5	10,1	28,2	61,3	0,2	15,7	57,8	26,3
12	0,9	7,8	9,4	81,9	1,8	8,8	11,1	78,3	1,9	18	19,2	60,9	1,7	34,3	36	28
13	0,3	19,3	26,4	54	0,3	24,9	28,4	46,4	0,2	24,1	24,6	51,2	3,9	20,9	23,8	51,4
average	**1**	**21**	**18**	**60**	**1**	**22**	**19**	**59**	**1**	**23**	**21**	**56**	**1**	**30**	**32**	**38**

Supplemetnal Methods

Mass spectrometry

For a detailed description please see supplemental methods in supplemental data. data Cells were cultivated for > 7 days in proline supplemented RPMI1640 medium for SILAC (Thermo Fisher Scientific) with conventional or heavy ($^{13}C_6$) L-arginine and ($^{13}C_6$) L-lysine (Thermo Fisher Scientific). “Heavy” labeled cells were stimulated with 20 µM MLN4924 overnight and “light” cells remained untreated. After three washes cells were pairwise combined and subjected to lysis in NuPAGE® LDS sample buffer (Life Technologies). Samples were reduced in with 50 mM DTT at 70 °C for 10 minutes and alkylated with 120 mM Iodoacetamide at room temperature for 20 minutes. Separation was performed on NuPAGE® Novex® 4-12 % Bis-Tris gels (Life Technologies) with MOPS buffer according to manufacturer’s instructions. Washed gels (3 x 5 min water) were stained for 1 h with Simply Blue™ Safe Stain (Life Technologies) and after washing with water for 1 h, each gel lane was cut into 15 slices. The slices were destained with 30 % acetonitrile in 0.1 M NH_4HCO_3 (pH 8), shrunk with 100 % acetonitrile, and dried in a vacuum concentrator (Concentrator 5301, Eppendorf, Germany). Samples were digested with 0.1 µg trypsin (overnight, 37 °C in 0.1 M NH_4HCO_3, pH 8). After removing the supernatant, peptides were extracted from the gel slices with 5 % formic acid, and pooled with the corresponding supernatant. NanoLC-MS/MS analyses were performed on an LTQ-Orbitrap Velos Pro (Thermo Scientific) equipped with an EASY-Spray Ion Source and coupled to an EASY-nLC 1000 (Thermo Scientific). Peptides were loaded on a trapping column (2 cm x 75 µm ID. PepMap C18, 3 µm particles, 100 Å pore size) and separated on an EASY-Spray column (25 cm x 75 µm ID, PepMap C18, 2 µm particles, 100 Å pore size) with a 120-minute linear gradient from 3% to 30% acetonitrile and 0.1% formic acid. MS scans were acquired in the Orbitrap analyzer with a resolution of 30,000 at m/z 400, MS/MS scans were acquired in the Ion Trap analyzer with normal scan rate using CID fragmentation with 35% normalized collision energy. A TOP10 data-dependent MS/MS method was used; dynamic exclusion was

applied with a repeat count of 1 and an exclusion duration of 120 seconds; singly charged precursors were excluded from selection. Minimum signal threshold for precursor selection was set to 20,000. AGC was used with AGC target with a value of 1e6 for MS scans and 1e4 for MS/MS scans. Lock mass option was applied for internal calibration in all runs using background ions from protonated decamethylcyclopentasiloxane (m/z 371.10124). For MS raw data file processing, database searches and quantification, MaxQuant version 1.4.1.2 was used[48]. The search was performed against the UniProt Human database. Additionally, a database containing common contaminants was used. Protein identification was under control of the false-discovery rate (<1% FDR on protein and peptide level). In addition to MaxQuant default settings, the search was performed with tryptic cleavage specificity with three allowed missed cleavages. The search was performed with the following variable modifications: Gln to pyro-Glu formation and oxidation (on Met). H/L ratios were used for protein quantitation (at least two peptides per protein).
